# Changes in Cell Aggregation in *Arabidopsis thaliana* Suspension Culture Following Knockout of GAUT Gene Family Members

**DOI:** 10.3390/plants14182816

**Published:** 2025-09-09

**Authors:** Tatyana A. Frankevich, Natalya V. Permyakova, Yury V. Sidorchuk, Elena V. Deineko

**Affiliations:** Federal Research Center Institute of Cytology and Genetics, Siberian Branch of Russian Academy of Sciences, pr. Lavrentieva 10, Novosibirsk 630090, Russia; puh@bionet.nsc.ru (N.V.P.); sidorch@bionet.nsc.ru (Y.V.S.); deineko@bionet.nsc.ru (E.V.D.)

**Keywords:** *A. thaliana*, cell culture, aggregation, genome editing, galacturonosyltransferase, GAUT7, Quasimodo1-1

## Abstract

Plant cells, particularly suspension cell cultures, represent a promising platform for the biosynthesis of biopharmaceutical proteins. However, one of the limitations of this system is the tendency of cells to form aggregates of varying sizes, which can restrict their productivity in terms of recombinant protein accumulation. The primary cause of such aggregation is likely related to intercellular adhesion, which is characteristic of plant cells grown in vitro. To assess the potential for reducing intercellular adhesion in in vitro plant cell cultures, we obtained two *Arabidopsis thaliana* cell lines carrying mutations in the *GAUT7* and *GAUT8* genes, which are involved in the biosynthesis of cell wall pectin. The *GAUT7* mutant was generated by CRISPR/Cas9-mediated knockout of the target gene in a transgenic *A. thaliana* line carrying the *gfp* gene. The GAUT8 mutant cell line was derived from the *A. thaliana* Quasimodo1-1 mutant. We present a comparative analysis of these two in vitro cultured cell lines in terms of their aggregation behavior. The resulting mutant with a knockout in the GAUT7 gene had an altered cell culture phenotype. The GAUT7 suspension culture was characterized by a darker coloration, an increase in the number of large aggregates by 18%, and a decrease in the level of pectins, and the accumulation of recombinant GFP protein in the GAUT7 culture significantly decreased by 10.4%. The Qua1-1 culture showed the opposite results: a 20% decrease in the number of large aggregates, a high increase in biomass, and an increased level of pectins compared with the control and GAUT7. Thus, we have shown that a violation of pectin synthesis leads to different results depending on which GAUT family gene we knock out; intercellular adhesion decreased in the cell culture with a knockout of the GAUT8 gene. These data can be used to improve the properties of plant cellular expression systems of biopharmaceutically valuable proteins.

## 1. Introduction

Plant cell cultures are widely used in studies of plant physiology and molecular biology, as they make it possible to investigate various processes while bypassing the complex organization of the whole plant. These cultures are characterized by a homogeneous cell population, rapid growth, the ability to obtain large amounts of biomass, and high reproducibility of cultivation conditions. All these features make them a convenient model system for the analysis of complex cellular and molecular mechanisms [1]. In addition to their scientific significance, plant cell cultures have practical applications—they are used as effective platforms for the production of secondary metabolites [2,3] and biopharmaceutically valuable proteins [4,5]. There are numerous examples of the successful use of plant cell cultures for the production of recombinant proteins: β-glucocerebrosidase produced in carrot cells [6], α1-antitrypsin (rAAT) in tobacco cells [7], and the cytokine hGM-CSF in rice cells [8]. However, one of the limitations of this platform is the ability of cells to form aggregates of various sizes, which may be one of the reasons for the still insufficient accumulation of recombinant proteins. It has been shown that reducing aggregation in plant cell cultures leads to an increase in the yield of secondary metabolites [9].

The main cause of cell aggregation in culture may be associated with intercellular adhesion, which is characteristic of plant cells. One of the main cell wall pectins, homogalacturonan (HG), plays a key role in this process and is encoded by genes of the GAUT family [10]. Suppression of *GAUT* family genes has already demonstrated positive effects on productivity: downregulation of the *GAUT4* gene led to a reduction in the content of pectin (homogalacturonan and rhamnogalacturonan II), which made it possible to increase the amount of released sugars in transgenic plants by a factor of 1.5 to 7, and ethanol production by 7 [11]. Similar results were observed upon downregulation of *GAUT12* [12].

The homogalacturonan pectin of the cell wall is polymerized by the GAUT1:GAUT7 enzymatic complex [13]. The *GAUT1* gene encodes the protein homogalacturonan α1,4-galacturonosyltransferase 1, which is the enzyme responsible for pectin biosynthesis. GAUT1 catalyzes the transfer of galacturonic acid from UDP-GalA to homogalacturonan during its polymerization [14,15]. We hypothesized that disruption of pectin biosynthesis caused by mutations in *GAUT1* and *GAUT7* may lead to changes in intercellular adhesion and, as a result, a reduction in aggregation in cell cultures induced from explants of such plants. Therefore, impairment of adhesion properties in in vitro cultures via alterations in pectin biosynthesis through the knockout of key genes may not only decrease cell aggregation but also enhance the accumulation of recombinant proteins.

However, our initial attempts to reduce intercellular adhesion in cell culture did not support this hypothesis. *A. thaliana* plants with a homozygous biallelic deletion in *GAUT1*, generated via CRISPR/Cas9-mediated genome editing, exhibited an altered phenotype and arrested development at early stages. Moreover, cell cultures induced from explants of these seedlings formed large aggregates [16]. Notably, increased aggregation in the *GAUT1*-knockout suspension culture was accompanied by a marked reduction in recombinant GFP accumulation. These findings indicated that to verify our hypothesis regarding the reduction of aggregation through pectin biosynthesis gene editing, other gene targets should be considered. We selected the *GAUT7* and *GAUT8* genes as alternative candidates. The *GAUT8* gene is also involved in homogalacturonan biosynthesis, as well as in the biosynthesis of xylan in the cell wall. The *GAUT8*-encoded protein in *A. thaliana* functions as an α-1,4-D-galacturonosyltransferase. A *T*-DNA insertional mutant in *GAUT8*, known as *Quasimodo1 (Qua1-1)*, has previously been described in the literature, where its role in affecting intercellular adhesion was demonstrated [17]. Unfortunately, the *Qua1-1* line cannot be used to assess GFP accumulation, since transgene insertion via Agrobacterium-mediated transformation occurs at random genomic locations, potentially leading to positional effects and variable expression levels of the *gfp* gene. Nevertheless, this line remains valuable for comparative analysis of cell culture characteristics.

Currently, one of the most effective strategies for generating high-yielding plant-based production systems is site-specific genome editing, particularly through the CRISPR/Cas9 system [18]. This system relies on a guide RNA (gRNA) containing a 20-nucleotide sequence complementary to the target genomic locus and the Cas9 endonuclease, which induces a double-strand break at the target site [19,20]. The subsequent repair process often results in small deletions or insertions that can inactivate the gene. CRISPR/Cas9-mediated gene editing has been successfully applied in a variety of plant species [21,22]. The simplicity and efficiency of Cas9 guided our choice for generating a *GAUT7* gene knockout.

Thus, the aim of this study was to investigate the roles of *GAUT7* and *GAUT8* in regulating cell aggregation in plant cell cultures.

## 2. Results

### 2.1. Plant Material Analysis

As a result of Agrobacterium-mediated transformation with the construct targeting the *GAUT7* gene, approximately 200 seeds exhibiting seed coat fluorescence were obtained. Seeds germinated efficiently both on soil and on sterile medium.

**Figure 1:**
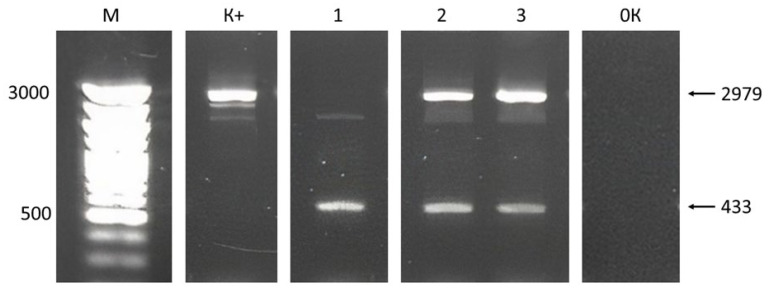
Electrophoresis of PCR products of plant DNA with a putative deletion in the *GAUT7* gene in 1.5% agarose gel. Designations: M—DNA marker Step 100 (Biolabmix, Novosibirsk, Russia), length in base pairs; K+—*GAUT7* gene fragment from the control Col-0 GFP line; 1, 2, and 3—*GAUT7* gene fragments; 0K—negative control; black arrows indicate gene target fragments with or without deletion, length in base pairs.

PCR screening of 96 individual T_0_ plants revealed 20 heterozygous lines carrying a deletion in one allele, and one homozygous line with a 2593 bp deletion in both alleles of the *GAUT7* gene. An example of PCR-based detection of deletions in genomic DNA from seedlings is shown in Figure 1. The presence of a single short amplicon (433 bp) in the electrophoresis gel indicates a homozygous mutation in the target gene, confirming deletions in both homologous chromosomes.

### 2.2. Phenotypic Characteristics of Callus and Suspension Cell Cultures with GAUT7 Knockout and the Qua1-1 Line

**Figure 2:**
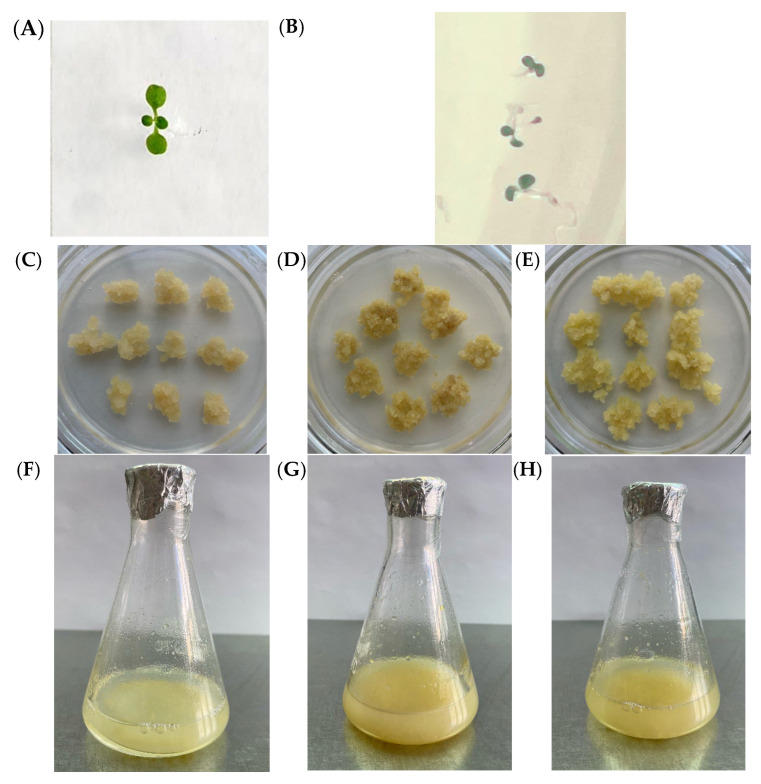
Phenotypes of *A. thaliana* Col-0 GFP, *GAUT7* knockout, and Qua1-1 (*GAUT8* knockout) lines. (**A**)—seedling of the control Col-0 GFP line; (**B**)—seedlings with *GAUT7* gene knockout; (**C**)—callus culture of Col-0 GFP; (**D**)—callus culture obtained from samples with *GAUT7* gene deletion; (**E**)—callus culture of Qua1-1; (**F**)—suspension culture of Col-0 GFP; (**G**)—suspension culture obtained from samples with *GAUT7* gene deletion; (**H**)—suspension culture of Qua1-1.

All resulting seedlings displayed a normal phenotype without visible morphological abnormalities and were indistinguishable from control plants (Figure 2A,B).

Callus and suspension cell cultures were established from seedlings homozygous for the deletion in the *GAUT7* gene. The resulting callus cultures were phenotypically different from the control callus cultures derived from unedited seedlings (Figure 2C). They exhibited a looser structure, likely due to impaired cell-to-cell adhesion, and appeared darker brown in color compared to the light-yellow control (Figure 2D).

Compared with the control culture (Figure 2F), suspension cell cultures obtained from GAUT7 knockout cells (Figure 2G) were phenotypically different. The culture obtained from GAUT7-mutant cells was darker in color, consisted of larger aggregates, and exhibited slower growth. The Qua1-1 line culture also displayed phenotypic differences from the control line. Both the callus and suspension cultures had a lighter color (Figure 2E,H, respectively). The Qua1-1 suspension culture visually contained minimal large aggregates, making it appear more homogeneous.

**Figure 3:**
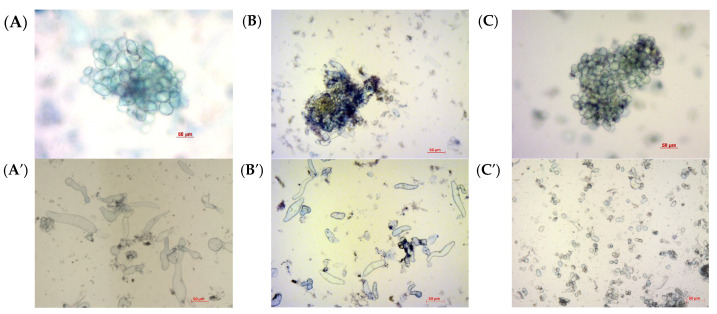
Micrographs of cells and cell aggregates in suspension cell cultures. (**A**,**A′**)—Col-0 GFP; (**B**,**B′**)—culture with deletion in the GAUT7 gene; (**C**,**C′**)—Qua1-1 culture. Scale bar: 50 μm.

Microscopic examination of the cells revealed changes in aggregate structure in the *GAUT7* knockout suspension cultures (Figure 3). In the control culture Col-0, cell aggregates were composed of clusters of cells connected at the center by cell walls and pectin (Figure 3A,A′). Lines carrying mutations in the *GAUT7* gene exhibited high aggregation; the central parts of aggregates were composed of dark green masses, likely composed of non-polymerized pectin. Small aggregates were rare, and the suspension contained few individual cells (Figure 3B,B′). The individual cells that were observed did not differ morphologically from the control (Figure 3A′,B′). In the Qua1-1 line, both cells and aggregates also had morphological differences from the Col-0 line including a rounded shape and cell sizes several times smaller, and the total number of single cells in suspension was significantly higher than in Col-0 and GAUT7 (Figure 3C,C′). At the same time, in the cell culture of the Qua1-1 line, the cells were evenly distributed in the aggregate.

**Figure 4:**
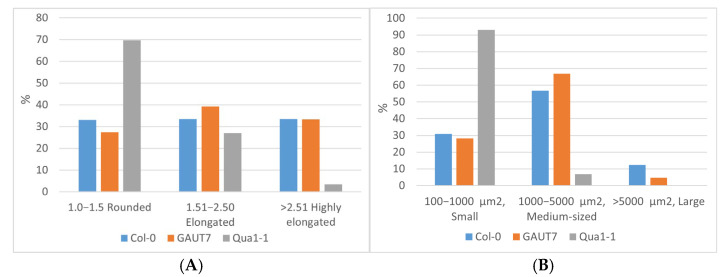
Cytological characterization of cell suspension cultures. (**A**)—Proportions of cells with different shapes in suspension cultures; (**B**)—proportions of cells of different sizes in suspension cultures.

Overall, when assessing the morphology of non-aggregated cells, approximately one third of the cells in suspensions induced from the calli of both the Col-0 and *GAUT7* lines exhibited a rounded, elongated, or highly elongated shape (Figure 4A). The proportions of cells of different sizes in these suspension cultures were also largely similar (Figure 4B). About 30% of the cells in both suspensions were small, while 56–67% had a medium size. A minor fraction consisted of large cells, with a higher proportion observed in the Col-0 suspension (12.3%), whereas in the *GAUT7* suspension, the proportion of large cells was 2.5 times lower (4.8%).

Individual cells and cell aggregates of the Qua1-1 line showed the most pronounced differences compared to both the control Col-0 line and the *GAUT7* line (Figure 4A,B). The vast majority (approximately 70%) of cells in the Qua1-1 suspension culture were rounded; the proportion of elongated cells was only slightly different from that in the Col-0 and *GAUT7* suspensions and amounted to 27.0%. However, the proportion of highly elongated cells was ten times lower than in Col-0 and *GAUT7* (Figure 4A). The most notable differences in this suspension culture were related to cell size: 93.1% of the cells were classified as small (Figure 4B). It is worth noting that while the minimum area of single cells in the Col-0 and *GAUT7* suspensions was around 250 µm^2^, in the Qua1-1 suspension culture this value started at approximately 150 µm^2^. The proportion of medium-sized cells in Qua1-1 was 8–10 times lower (6.9%) than in Col-0 and *GAUT7*, and the class of large cells was completely absent (Figure 4B).

### 2.3. Biomass Accumulation and Aggregation of Suspension Cultures

**Figure 5:**
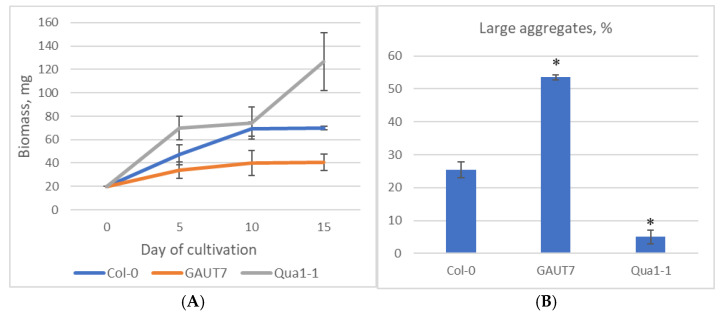
Results of dry biomass accumulation analysis (**A**) and aggregation analysis (**B**) in suspension cell cultures. Designations: Col-0—suspension culture derived from Col-0 GFP line cells; GAUT7—suspension culture derived from cells homozygous for the *GAUT7* gene deletion; Qua1-1—Qua1-1 suspension culture. Note: *—Statistically significant differences from the control within the corresponding line according to the Kruskal–Wallis test at *p* ≤ 0.05.

The results of comparative analysis of growth characteristics and aggregation in suspension cultures of the three cell lines—Col-0 GFP, GAUT7, and Qua1-1—are presented in Figure 5. Biomass accumulation analysis showed no statistically significant differences between the control and the GAUT7 mutant line according to the Kruskal–Wallis test (*p* ≤ 0.05), whereas the Qua1-1 line accumulated significantly more biomass than the control and exhibited statistically significant differences (Figure 5A).

The aggregation analysis, based on the comparison of the proportions of small (≤1 mm in diameter) and large aggregates (>1 mm in diameter) and evaluated using the Kruskal–Wallis test (*p* ≤ 0.05), demonstrated that *GAUT7* mutants accumulated 18% more large aggregates than the control Col-0 suspension culture (Figure 5B). Thus, the *GAUT7* mutation did not result in increased biomass accumulation compared to the parental line, but did lead to a higher proportion of large aggregates. In contrast, the Qua1-1 line showed a high increase in biomass along with a statistically significant 20% reduction in the proportion of large aggregates relative to the Col-0 suspension culture (Figure 5B). It is likely that the mutation in the *GAUT8* gene reduces pectin polymerization in a way that does not interfere with cell division and promotes the formation of small aggregates, from which individual cells can easily separate. These findings are consistent with the results of microscopic examination of the suspension cultures.

### 2.4. Pectin Content Analysis in the Cell Wall

**Figure 6:**
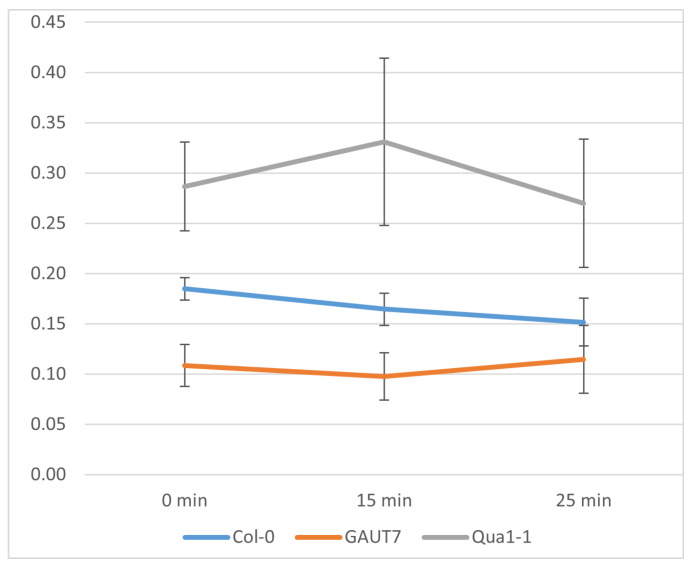
Quantification of pectin content in three cell lines: Col-0 GFP, GAUT7, and Qua1-1.

The results of pectin analysis in suspension cultures of the three cell lines—Col-0 GFP, GAUT7, and Qua1-1—are presented in Figure 6.

Pectin quantification analysis revealed statistically significant differences according to the Kruskal–Wallis test (*p* ≤ 0.05). The Qua1-1 mutants contained higher levels of pectin compared to both the Col-0 and GAUT7 lines, whereas the *GAUT7* mutant exhibited significantly lower pectin content than Col-0 and Qua1-1.

### 2.5. Analysis of Recombinant GFP Protein Accumulation

Quantitative analysis of GFP protein levels in mutant and control lines (Table 1) showed that the GAUT7 mutant line accumulated significantly less GFP than the control. These results are consistent with the observed changes in aggregation: *GAUT7* knockout mutants formed a greater proportion of large aggregates, which in turn reduced the yield of recombinant protein.

## 3. Discussion

It is known that genes of the GAUT family can impair cell adhesion by altering homogalacturonan synthesis [13,14]. This pectin is polymerized by the enzymatic GAUT1:GAUT7 complex [14]. The *GAUT1* gene encodes homogalacturonan α1,4-galacturonosyltransferase 1, whereas *GAUT7* anchors this enzyme to the Golgi membrane. The GAUT1 enzyme, encoded by the *GAUT1* gene, is a key enzyme responsible for the synthesis of pectins. However, our previous study [16] demonstrated that knockout of the *GAUT1* gene has a negative impact on cell culture, paradoxically increasing cell aggregativity and, consequently, reducing the accumulation of the target recombinant protein. The protein encoded by *GAUT7* plays a less central role in pectin polymerization compared to *GAUT1*. Therefore, we hypothesized that *GAUT7* knockout would have milder effects than *GAUT1* knockout. Another gene important for pectin biosynthesis within the GAUT family is *GAUT8*, whose protein product shares 77% amino acid identity with GAUT1 [23]. In *A. thaliana* Qua1-1 heterozygous mutants, reduced adhesion of leaf epidermal cells and root tip cells has been observed [24], leading to dwarfism, while homozygous mutants often exhibit a lethal phenotype [23]. The GalA content in the cell walls of these mutants is reduced by 25%. Additionally, membrane fractions from the stem tissue of Qua1-1 mutants show reduced activities of both α1,4-GalAT and β1,4-XylT [25]. This suggests that *GAUT8* may participate in the synthesis of both homogalacturonan and xylan in the plant cell wall.

Seeds obtained after the transformation of plants with a construct designed to knock out the *GAUT7* gene did not differ in germination rate from control plants, and the resulting plants showed no morphological abnormalities. Thus, unlike *GAUT1* knockout, which resulted in the formation of non-viable plants [16], the *GAUT7* gene does not have a significant impact on the whole-plant phenotype compared to the control. This could be due to genetic redundancy, as other genes such as *GAUT5* and *GAUT6* may partially compensate for the function of *GAUT7*. Although GAUT7 single mutants do not typically show strong phenotypic alterations, combinations with other mutants (e.g., GAUT5 and GAUT6) have been associated with changes in pollen tube length [26].

Until now, the *GAUT7* mutant phenotype has only been analyzed at the whole-plant level. Although the knockout does not affect whole-plant development, our results show that specific changes occur at the cell culture stage. GAUT7 knockout cell cultures—including both callus and suspension cultures—differ from the control: they exhibit a darker coloration and a looser structure. Cell darkening may result from various stress conditions such as nutrient deficiency, light stress, or accumulation of phenolic compounds and secondary metabolites [27,28]. While *GAUT5* and *GAUT6* may compensate for *GAUT7*’s function in planta, their expression patterns differ, and compensation may not occur under in vitro culture conditions. Moreover, the effect may be enhanced in cell cultures due to altered hormone signaling and the absence of defined cell orientation, unlike in organized plant tissues.

In the Col-0 and *GAUT7* lines, cell populations in culture are predominantly composed of elongated, often cylindrical cells of medium and large size. In contrast, the Qua1-1 culture is characterized by a pronounced prevalence of small, rounded cells. This suggests that cells in Col-0 and GAUT7 cultures progress more rapidly through the exponential growth phase, predominantly via cell elongation [29]. By the same cultivation stage, Qua1-1 cells are also in exponential growth, likely due to differences in initial cell density. A finely aggregated culture with a high number of single cells has better access to nutrients and is less prone to necrosis, thereby providing more favorable conditions for growth, development, and accumulation of secondary metabolites [9,30].

According to pectin measurements, the finely aggregated Qua1-1 culture accumulates greater biomass, which results in a higher level of pectin accumulation.

One possible explanation for the changes in aggregate sizes in mutants of the *GAUT7* and *GAUT8* genes may lie in the disruption of methyl-esterification of homogalacturonan in these mutants, which is associated with impaired formation of calcium cross-links in the cell wall, affecting its rigidity [31,32]. Moreover, changes in homogalacturonan methyl-esterification can have several underlying reasons—either improperly formed pectins are more reactive and interact more readily with calcium ions, or in mutants of the *GAUT7* and *GAUT8* genes, the activity of pectin methylesterase is compensatorily altered. However, since we observe opposite effects in different mutants, it appears that the primary factor is the disruption of pectin formation itself.

Nutrient deficiency and cellular stress, apparently, also contribute to the observed reduction in recombinant protein accumulation in the *GAUT7* knockout cell culture.

Understanding the aggregation process mediated by *GAUT* genes can find practical application in plant biotechnology, for example, for the production of secondary metabolites or biopharmaceutically valuable proteins. Future research will focus on expanding work on these genes: testing double knockouts, using complementary lines, analyzing the composition of secreted pectin, and studying the biochemical relationships between HG and other components of the cell wall. Based on these findings, future work will involve inactivation of *GAUT8* in the Col-0 GFP producer line to evaluate the effect of *GAUT8* knockout on culture productivity. Other promising target genes for future study include less-characterized members of the GAUT family such as *GAUT9*, *GAUT10*, and *GAUT11*. In addition, knockout of genes encoding rhamnogalacturonan I and II (RGTX1 and RGTX2)—key pectic components of the plant cell wall—is also under consideration.

## 4. Materials and Methods

### 4.1. Plant Materia

The initial plant material used for *GAUT7* gene knockout consisted of transgenic *A. thaliana* (L.) Heynh. (ecotype Columbia-0) plants from a homozygous line containing a single copy of the *gfp* gene. This parental line was previously generated in our laboratory via Agrobacterium-mediated transformation and random integration of a transgene construct. The integration site was later mapped to the 3′ untranslated region of gene AT4G39600 (unpublished data). Seeds of the Qua1-1 line, which carries a T-DNA-induced mutation in the *GAUT8* gene, were kindly provided by Dr. Stéphane Verger from the Department of Forest Genetics and Plant Physiology at the Swedish University of Agricultural Sciences [33].

### 4.2. Plasmids Carrying Cas9 and Guide RNAs

Plasmids pDGE332 (Addgene №153241) and pDGE334 (Addgene №153243) were used as intermediates in the cloning workflow, while pDGE347 (Addgene №153228), containing the *Cas9* endonuclease gene driven by the *A. thaliana RPS5a* promoter, served as the final expression vector. These plasmids were generously provided by J. Stuttmann [34]. For plant selection, the constructs included the *FAST* marker—a fusion of the oleosin gene and the gene encoding RFP, enabling red fluorescence in the seed coat and the *bar* gene, which confers resistance to the herbicide phosphinothricin.

### 4.3. Guide RNA Selection

To disrupt the target gene, two gRNA target sites were selected to induce a deletion of a substantial genomic fragment. Candidate gRNA sequences were identified using the CRISPOR v.5.2 [35] and CRISPR-P v2.0 [36] online tools. RNA secondary structures were analyzed using the RNAfold web server. The oligonucleotide sequences used for the assembly of the gRNA cassettes are listed in Table 2.

### 4.4. Construction of the pDGE347_GAUT7 Genetic Construct

**Figure 7:**
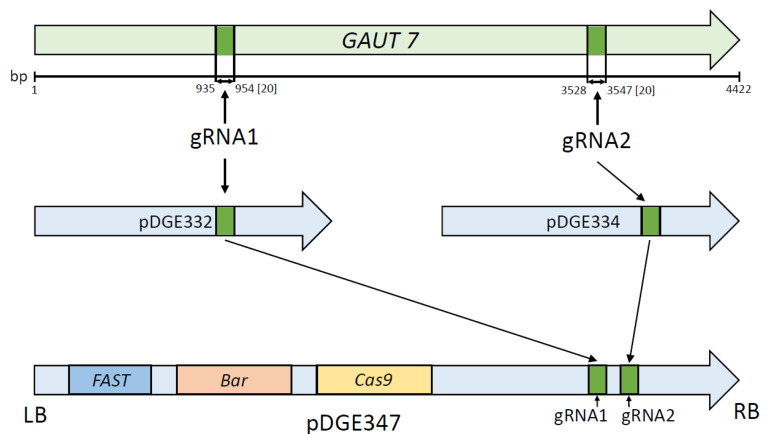
Schematic representation of the genetic construct assembly for GAUT7 gene knockout. Designations: *GAUT7*—target gene, the locations of the editing sites, and the distances (in bp) from the start codon are indicated; LB and RB—left and right T-DNA border repeats; gRNA1/gRNA2—guide RNA target sequences homologous to the selected regions of the *GAUT7* gene; pDGE332/pDGE334—intermediate cloning vectors; pDGE347—final binary vector used for plant transformation; *FAST*—marker gene conferring red fluorescence to the seed coat of transgenic seeds; *bar*—gene encoding phosphinothricin N-acetyltransferase, conferring resistance to phosphinothricin; *Cas9*—gene encoding the Cas9 endonuclease.

The selected gRNA sequences were transferred into the pDGE347 plasmid using the intermediate vectors pDGE332 and pDGE334 for the first and second target sites, respectively. The initial step in the assembly process involved the annealing of phosphorylated oligonucleotides and their subsequent insertion into the pDGE332 and pDGE334 plasmids via Golden Gate cloning using the BbsI restriction enzyme (№R0539S, New England Biolabs, Ipswich, MA, USA). In the second step, the resulting sgRNAs carrying the guide sequences were transferred into the pDGE347 backbone. This was accomplished through Golden Gate assembly using the BsaI-HF restriction enzyme (№R3733S, New England Biolabs, Ipswich, MA, USA). A schematic overview of the cloning strategy is shown in Figure 7. To confirm the successful insertion of the target sequences, plasmid DNA from the resulting clones was sequenced using the primers pDGEtest up and pDGEtest lo (Table 2). Sanger sequencing was performed by the company Evrogen (Moscow, Russia).

### 4.5. Delivery of the Genetic Construct into Plants

*Agrobacterium tumefaciens* strain GV3101 was used for transformation via the floral dip method [37]. Transgenic seeds were selected based on red fluorescence of the seed coat under blue light illumination using a fluorophore detection lamp (Dark Reader Hand Lamp, HL34T, Clare Chemical Research, Dolores, CO, USA).

### 4.6. Analysis of Transformants for GAUT7 Deletion

Fluorescent seeds were sown on soil and sterile Murashige and Skoog (MS) medium [38]. Prior to sowing, seeds were sterilized by treatment with 4% H_2_O_2_ for 1 min. Genomic DNA was extracted from the resulting seedlings according to the protocol by Kasajima [39], and PCR analysis was performed to detect deletions in the *GAUT7* gene using the primers GAUT7_deltest_Up2 and GAUT7_deltest_Lo2 (Table 2). PCR was carried out using Blitz polymerase (BelbioLab, Moscow, Russia).

PCR reaction: H_2_O—6.6 µL, DNA—1 µL, Blitz 2.5x buffer—8 µL, DelUp and DelLo 10x primers—2 µL, Blitz polymerase—0.4 µL. Annealing 55°, elongation 1.5 min.

### 4.7. Establishment of Suspension Cell Cultures

Callus cultures were induced from seedlings carrying deletions in both alleles of the *GAUT7* gene, as well as from Qua1-1 line seedlings, by transferring explants onto solid SH medium [40]. Suspension cultures were subsequently initiated by transferring callus tissue into liquid SH medium [40].

### 4.8. Analysis of Biomass Accumulation and Aggregation in Suspension Cultures

To compare growth performance, biomass accumulation was assessed alongside an analysis of aggregation behavior in suspension cultures. As a control, suspension cultures derived from unedited parental cell lines containing the *gfp* gene were used. For biomass assessment, 3 mL of suspension culture was transferred into 20 mL of liquid SH medium. On days 0, 5, 10, and 15 of cultivation, dry filters were weighed, and small and large aggregates were separated using a 1 mm mesh filter. The biomass retained on the filters was dried at room temperature for 3 days. The net biomass was calculated by subtracting the mass of the dry filter from the total. The total culture biomass was determined by summing the dry weights of small and large aggregates.

### 4.9. Analysis of Pectin Content in the Cell Wall

Pectin content was analyzed in three biological replicates using the Pectin Identification Assay Kit (Megazyme, Bray, Ireland). Absorbance measurements were performed using a SmartSpec Plus spectrophotometer (Bio-Rad, Hercules, CA, USA).

### 4.10. Light Microscopy

Morphological analysis of aggregates in suspension cultures was conducted using light microscopy. Samples were stained with trypan blue [41]. All images were obtained using an AxioImager Z1 microscope (Zeiss, Jena, Germany) at 100× magnification with an AxioCam MRm camera.

Cytological characterization of cell suspension cultures was carried out by calculating and measuring cell indices and cell area using micrographs in the ZEISS ZEN program. The sample of cells consisted of 40 cells in 9 replicates for each line: Col-0, GAUT7, and Qua1-1.

### 4.11. Quantification of Recombinant GFP Protein

To evaluate recombinant protein accumulation, GFP fluorescence intensity was measured in extracts from 300 mg of suspension cell biomass on day 7 of cultivation [42]. To normalize the samples, total protein content was also determined using the Bradford method [43]. Measurements were performed on a CLARIOstar Plus multimode reader (BMG LABTECH, Ortenberg, Germany).

### 4.12. Statistical Analysis

Statistical analysis was performed using the STATISTICA 10 software. A non-parametric one-way ANOVA was conducted using the Kruskal–Wallis test, followed by the post hoc Dunn’s test. Differences between groups were considered statistically significant at a *p*-value < 0.05.

## Figures and Tables

**Table 1:** Quantitative analysis of GFP protein content in *A. thaliana* suspension cultures.

Suspension Cell Culture Line	Total Soluble Protein, mg/μL	GFP Protein, mg/μL	GFP as a Percentage of Total Soluble Protein, %
Col-0 GFP	0.40	0.063	16
GAUT7	0.96	0.053	5.6 *

Note: *—Statistically significant difference from the control within the same line according to the Kruskal–Wallis test at *p* ≤ 0.05.

**Table 2:** Oligonucleotides used in this study.

Oligonucleotide Name	Nucleotide Sequence 5′–3′
GAUT7_gRNA3_Forward	ATTGAATCAATCCAGTTCTTCCCA
GAUT7_gRNA3_Reverse	AAACTGGGAAGAACTGGATTGATT
GAUT7_gRNA4_Forward	ATTGCTTCCATATCAAGGTCCCAA
GAUT7_gRNA4_Reverse	AAACTTGGGACCTTGATATGGAAG
GAUT7_deltest_Up2	TGTCACTGTTCAACCGGCTTCTT
GAUT7_deltest_Lo2	CAATGCCCTCCATCTAGCAAGATC
pDGEtest up	ATAGCAATGACCAGTGCAAACAGTG
pDGEtest lo	CTCTTTTCTCTTAGGTTTACCCGCC

## Data Availability

The original contributions presented in this study are included in the article. Further inquiries can be directed to the corresponding author.

## References

[1] Cimini S., Ronci M.B., Barizza E., de Pinto M.C., Locato V., Lo Schiavo F., De Gara L. (2018). Plant cell cultures as model systems to study programmed cell death. Plant Program. Cell Death Methods Protoc..

[2] Tabata H. (2006). Production of paclitaxel and the related taxanes by cell suspension cultures of Taxus species. Curr. Drug Targets.

[3] Chattopadhyay S., Datta S.K., Mahato S.B. (1994). Production of L-DOPA from cell suspension culture of *Mucuna pruriens f. pruriens*. Plant Cell Rep..

[4] Bapat V.A., Kavi Kishor P.B., Jalaja N., Jain S.M., Penna S. (2023). Plant cell cultures: Biofactories for the production of bioactive compounds. Agronomy.

[5] Zagorskaya A.A., Deineko E.V. (2017). Suspension-cultured plant cells as a platform for obtaining recombinant proteins. Russ. J. Plant Physiol..

[6] Grabowski G.A., Golembo M., Shaaltiel Y. (2014). Taliglucerase alfa: An enzyme replacement therapy using plant cell expression technology. Mol. Genet. Metab..

[7] Huang T.K., Plesha M.A., Falk B.W., Dandekar A.M., McDonald K.A. (2009). Bioreactor strategies for improving production yield and functionality of a recombinant human protein in transgenic tobacco cell cultures. Biotechnol. Bioeng..

[8] Shin Y.J., Hong S.Y., Kwon T.H., Jang Y.S., Yang M.S. (2003). High level of expression of recombinant human granulocyte-macrophage colony stimulating factor in transgenic rice cell suspension culture. Biotechnol. Bioeng..

[9] Kolewe M.E., Henson M.A., Roberts S.C. (2011). Analysis of aggregate size as a process variable affecting paclitaxel accumulation in Taxus suspension cultures. Biotechnol. Prog..

[10] Daher F.B., Braybrook S.A. (2015). How to let go: Pectin and plant cell adhesion. Front. Plant Sci..

[11] Biswal A.K., Atmodjo M.A., Li M., Baxter H.L., Yoo C.G., Pu Y., Lee Y.C., Mazarei M., Black I.M., Zhang J.Y. (2018). Sugar release and growth of biofuel crops are improved by downregulation of pectin biosynthesis. Nat. Biotechnol..

[12] Biswal A.K., Hao Z., Pattathil S., Yang X., Winkeler K., Collins C., Mohanty S.S., Richardson E.A., Gelineo-Albersheim I., Hunt K. (2015). Downregulation of GAUT12 in Populus deltoides by RNA silencing results in reduced recalcitrance, increased growth and reduced xylan and pectin in a woody biofuel feedstock. Biotechnol. Biofuels.

[13] Atmodjo M.A., Sakuragi Y., Zhu X., Burrell A.J., Mohanty S.S., Atwood J.A., Orlando R., Scheller H.V., Mohnen D. (2011). Galacturonosyltransferase (GAUT) 1 and GAUT7 are the core of a plant cell wall pectin biosynthetic homogalacturonan: Galacturonosyltransferase complex. Proc. Natl. Acad. Sci. USA.

[14] Atmodjo M.A., Hao Z., Mohnen D. (2013). Evolving Views of Pectin Biosynthesis. Annu. Rev. Plant Biol..

[15] Sterling J.D., Atmodjo M.A., Inwood S.E., Kumar Kolli V.S., Quigley H.F., Hahn M.G., Mohnen D. (2006). Functional identification of an *Arabidopsis* pectin biosynthetic homogalacturonan galacturonosyltransferase. Proc. Natl. Acad. Sci. USA.

[16] Frankevich T.A., Permyakova N.V., Sidorchuk Y.V., Deineko E.V. (2025). Impact of GAUT1 Gene Knockout on Cell Aggregation in *Arabidopsis thaliana* Suspension Culture. BioTech.

[17] Leboeuf E., Guillon F., Thoiron S., Lahaye M. (2005). Biochemical and immunohistochemical analysis of pectic polysaccharides in the cell walls of *Arabidopsis* mutant QUASIMODO 1 suspension-cultured cells: Implications for cell adhesion. J. Exp. Bot..

[18] Jiang F., Doudna J.A. (2017). CRISPR–Cas9 structures and mechanisms. Annu. Rev. Biophys..

[19] Bortesi L., Zhu C., Zischewski J., Perez L., Bassié L., Nadi R., Forni G., Lade S.B., Soto E., Jin X. (2016). Patterns of CRISPR/Cas9 activity in plants, animals and microbes. Plant Biotechnol. J..

[20] Khatodia S., Bhatotia K., Passricha N., Khurana S.M., Tuteja N. (2016). The CRISPR/Cas genome-editing tool: Application in improvement of crops. Front. Plant Sci..

[21] Arora L., Narula A. (2017). Gene editing and crop improvement using CRISPR-Cas9 system. Front. Plant Sci..

[22] Demirci Y., Zhang B., Unver T. (2018). CRISPR/Cas9: An RNA-guided highly precise synthetic tool for plant genome editing. J. Cell. Physiol..

[23] Caffall K.H., Pattathil S., Phillips S.E., Hahn M.G., Mohnen D. (2009). *Arabidopsis thaliana* T-DNA mutants implicate GAUT genes in the biosynthesis of pectin and xylan in cell walls and seed testa. Mol. Plant.

[24] Durand C., Vicré-Gibouin M., Follet-Gueye M.L., Duponchel L., Moreau M., Lerouge P., Driouich A. (2009). The organization pattern of root border-like cells of *Arabidopsis* is dependent on cell wall homogalacturonan. Plant Physiol..

[25] Bouton S., Leboeuf E., Mouille G., Leydecker M.T., Talbotec J., Granier F., Lahaye M., Höfte H., Truong H.N. (2002). QUASIMODO1 encodes a putative membrane-bound glycosyltransferase required for normal pectin synthesis and cell adhesion in *Arabidopsis*. Plant Cell.

[26] Lund C.H., Stenbæk A., Atmodjo M.A., Rasmussen R.E., Moller I.E., Erstad S.M., Biswal A.K., Mohnen D., Mravec J., Sakuragi Y. (2020). Pectin synthesis and pollen tube growth in *Arabidopsis* involves three GAUT1 Golgi-anchoring proteins: GAUT5, GAUT6, and GAUT7. Front. Plant Sci..

[27] Xie J., Qi B., Mou C., Wang L., Jiao Y., Dou Y., Zheng H. (2022). BREVIPEDICELLUS and ERECTA control the expression of AtPRX17 to prevent *Arabidopsis* callus browning. J. Exp. Bot..

[28] Qiu L., Su J., Fu Y., Zhang K. (2023). Genetic and Transcriptome Analyses of Callus Browning in Chaling Common Wild Rice (*Oryza rufipogon* Griff.). Genes.

[29] Santos R.B., Abranches R., Fischer R., Sack M., Holland T. (2016). Putting the spotlight back on plant suspension cultures. Front. Plant Sci..

[30] Patil R.A., Kolewe M.E., Roberts S.C. (2013). Cellular aggregation is a key parameter associated with long term variability in paclitaxel accumulation in Taxus suspension cultures. Plant Cell. Tissue Organ Cult..

[31] Hepler P.K., Winship L.J. (2010). Calcium at the cell wall-cytoplast interface. J. Integr. Plant Biol..

[32] Wdowiak A., Podgórska A., Szal B. (2024). Calcium in plants: An important element of cell physiology and structure, signaling, and stress responses. Acta Physiol. Plant..

[33] Verger S., Chabout S., Gineau E., Mouille G. (2016). Cell adhesion in plants is under the control of putative O-fucosyltransferases. Development.

[34] Stuttmann J., Barthel K., Martin P., Ordon J., Erickson J.L., Herr R., Ferik F., Kretschmer C., Berner T., Keilwagen J. (2021). Highly efficient multiplex editing: One-shot generation of 8× Nicotiana benthamiana and 12× *Arabidopsis* mutants. Plant J..

[35] Concordet J.P., Haeussler M. (2018). CRISPOR: Intuitive guide selection for CRISPR/Cas9 genome editing experiments and screens. Nucleic Acids Res..

[36] Liu H., Ding Y., Zhou Y., Jin W., Xie K., Chen L.L. (2017). CRISPR-P 2.0: An improved CRISPR-Cas9 tool for genome editing in plants. Mol. Plant.

[37] Zhang X., Henriques R., Lin S.S., Niu Q.W., Chua N.H. (2006). Agrobacterium-mediated transformation of *Arabidopsis thaliana* using the floral dip method. Nat. Protoc..

[38] Murashige T., Skoog F. (1962). A revised medium for rapid growth and bio assays with tobacco tissue cultures. Physiol. Plant..

[39] Kasajima I., Ide Y., Ohkama-Ohtsu N., Hayashi H., Yoneyama T., Fujiwara T. (2004). A protocol for rapid DNA extraction from *Arabidopsis thaliana* for PCR analysis. Plant Mol. Biol. Report..

[40] Schenk R.U., Hildebrandt A.C. (1972). Medium and techniques for induction and growth of monocotyledonous and dicotyledonous plant cell cultures. Can. J. Bot..

[41] Strober W. (2015). Trypan blue exclusion test of cell viability. Curr. Protoc. Immunol..

[42] Wu J.J., Liu Y.W., Sun M.X. (2011). Improved and high throughput quantitative measurements of weak GFP expression in transgenic plant materials. Plant Cell Rep..

[43] Bradford M.M. (1976). A rapid and sensitive method for the quantitation of microgram quantities of protein utilizing the principle of protein-dye binding. Anal. Biochem..

